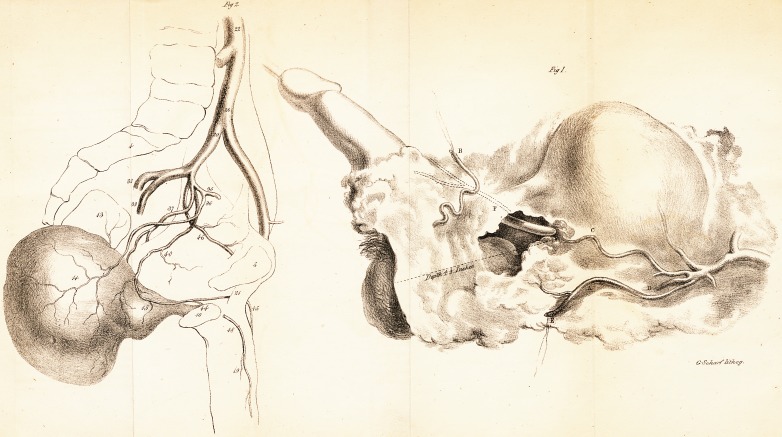# Case of Lithotomy, Attended with Hemorrhage

**Published:** 1826-01

**Authors:** John Shaw

**Affiliations:** Surgeon to the Middlesex Hospital, &c.


### ORIGINAL COMMUNICATIONS,

### SELECT OBSERVATIONS, &c.

---

**ART. I.—*Case of Lithotomy, attended with Hemorrhage.* By JOHN SHAW, Esq. Surgeon to the Middlesex Hospital, &c.**

[With an Engraving.]

*To the Editors of the London Medical and Physical Journal.*

GENTLEMEN,—The subject of hemorrhage after the operation of lithotomy is considered of such importance, that I am induced to believe the history of the following case will be acceptable to many of your readers.

JOHN SHAW.

The patient was a stout and fat country man, about sixty years of age. When he came to the hospital, he did not know the nature of his complaint: indeed, so little idea had he that the cause of his symptoms was a stone in the bladder, that, on my proposing to sound him, he left the hospital. He was, however, persuaded to return; and, about ten days after his admission, he submitted to be sounded. As the rub of the stone was sometimes felt only on the entry of the staff through the neck of the bladder, I was led to suspect that it was either a calculus projecting from a bed in the prostate, and which was pushed back into its sac by the convexity of the staff going over it, or that it was so small that it might, perhaps, be passed by the urethra.

Under these circumstances, and as the slight irritation under which the patient suffered was always easily relieved, I did not perform the operation until he had been some time in the hospital, and had been repeatedly sounded.

*Operation.*—After marking the place of the tuberosities of the ischia, I passed my left forefinger into the rectum, for the purpose of guarding the gut during the first steps of the operation. I commenced my incision an inch above the anus, near the raphé, and carried it obliquely past the anus. The patient being very fat, I at once thrust the scalpel to a considerable depth; so that this incision went through the skin, and at least an inch of fat. After cutting through more of this adipose substance, I withdrew my finger from the rectum, and passed it into the wound, for the purpose of directing the incisions through the levator ani. Then, feeling the staff through the face of the prostate, I cut upon it, and carried my knife forward through the membranous part of the urethra and prostate, following and directing the point with the forefinger of my left hand. On making this incision, there was a gush of fluid: I thought that it was urine; but, on observing it flowing over the back of my left hand, I saw that it was blood. I, however, proceeded with the operation; and, on the bladder being fairly cut into, there was a second gush of fluid, which was urine. Before introducing the forceps, I enlarged the wound a little, with a common curved bistoury. I now felt a very small stone lying on the tip of my forefinger: this was easily brought away by the common forceps; and, on introducing the finger again, a second stone, of a similar size, was felt, which was as easily extracted. The operation, I am told, did not take up more than three or four minutes.

The blood now flowed so profusely, that I was at first afraid I had wounded the pudic; and I, therefore, immediately put my finger on the place of that artery. I was relieved by finding it beating distinctly and strongly; but, though pressure was made on it by me and my colleagues, the bleeding was not at all commanded. Now, recollecting that the bleeding had taken place immediately on my cutting through the prostate, and considering that the pudic artery was felt distinctly, while pressure on it did not stop the hemorrhage, I hoped that the bleeding, although of an arterial colour, might be principally from the veins of the prostate. The patient was kept on the table for a considerable time, when, the bleeding appearing to subside, he was carried to bed. For the first ten minutes, the lower part of his body was left exposed, and his thighs and perineum were covered with wet towels: but, it being found, on examining the sheet, that he had lost about six ounces of blood, the wound was again examined.

It happened to be a very bright sunshine, and the patient's bed being close by a window having a south-west exposure, it was possible to place him so that the sun shone fairly into the wound, when the lips were held open. The hemorrhage had now so far subsided, that the wound could be sponged clean to such a depth that the place of the trunk of the pudic could be seen: but, as this vessel was distinctly felt beating all along the ramus, and as pressure on it had still no effect on the bleeding, we were satisfied that it was untouched; and the more especially as, by sponging the wound clean, we could *see* the blood oozing apparently from the bladder. But as, from the depth of the parts, it was not possible to apply a ligature with any degree of certainty,* and as there was now only an oozing, a

---

* On examination after death, the distance from the external wound to the source of the hemorrhage was found to be four and a half inches. See explanation of the Plate.

canula, with lint and sponge wrapped round it, was introduced into the neck of the bladder.

After this there was not any more bleeding, excepting a little through the canula, which soon stopped of itself. It was not thought proper to plug the canula, as the bladder might have been filled.

About four o'clock, the patient became so restless, and begged so much to be allowed to pass his urine, that I suspected the bladder might be filled with coagula, and I therefore removed the canula; but, on putting my finger into the bladder, I found very little blood there. As there was now no hemorrhage, the tube was not again introduced.

The patient presently began to complain very much of pain in his abdomen and chest. Being cold and the pulse low, he got some cordials; after which, he was more composed; but, from seven to nine, he seemed to suffer excruciating pain in his bowels. This increased so much, that he could not lie still: indeed, he got up several times, and was with difficulty restrained from getting out of bed; but it was remarkable that, notwithstanding these struggles, he never fainted: indeed, the loss of blood did not seem to have weakened him. During this time he occasionally took a table-spoonful of brandy, with a little wine and sugar. From nine to ten, he was more composed: after this, he again seemed to suffer excessively from spasm; and he died about half-past eleven.

It may be supposed that the symptoms just detailed are not those consequent on the loss of blood, and that they are more like the symptoms of inflammation of the bowels. It is not easy to explain the cause of the violent pain in the chest and abdomen which follows the loss of blood; but I believe the fact of its frequent occurrence is so well established, that few surgeons would be deceived by it so as to treat a patient under these circumstances for inflammation of the bowels.

On the following morning, the body was injected; and this was done by separate pipes in both common iliac arteries, the two external iliacs being tied. The inferior mesenteric artery was also injected by another pipe. My reason for proceeding in this way, was to insure the injection passing through every vessel that had been wounded. It is well known to all practical anatomists that, when it is attempted to inject the arteries of the pelvis with wax by one pipe put into the aorta, many of the small vessels are not filled. The inferior mesenteric was injected, because (as I had, during the operation, felt a large artery beating in the rectum,) I thought it possible that a branch from it might have been the vessel wounded.

The vessels in the perineum were first examined; and, after it was found that the trunk of the pudic and the artery of the bulb were entire, the penis, bladder, and rectum were taken out; the muscles and arteries being cut off close from the rami of the ischia.

The first figure in the Plate, which is taken from a drawing made from the parts after I had demonstrated them to the pupils, and had shown them to several of my friends, will sufficiently explain the source of the bleeding.

The original drawing is nearly of the full size; the depth from the external wound to that in the prostate being four inches and a half.

A is the trunk of the Internal Pudic. This artery, in separating the parts from the ramus of the ischium, was necessarily divided at E.

B, is the Artery of the Bulb, raised out of its natural situation, and held up by a thread. This artery was untouched. In this view it necessarily appears separated from the main pudic: but the circumstance of its being completely distended with injection is sufficient proof that it could not have been wounded during the operation.

D, is the Ischiatic, cut off short.

C, is a vessel of considerable magnitude, imperfectly filled with wax as far as the margin of the wound through the prostate.

The dotted lines, F, mark the continuation of the same vessel, passing under the pubes into the body of the penis. This part of the vessel is represented in outline; the wax from C having escaped into the bladder.

By making the examination of the vessels in this way, there is a proof, as far as dissection will afford, that the vessel C was wounded in cutting through the prostate. It may also be assumed, that the several circumstances of there being a gush of blood at the moment the prostate was cut,—that pressure on the main pudic against the ramus of the ischium had no influence over the hemorrhage,—and that the blood was *seen* to ooze from the neck of the bladder, are all evidences that the wound in the vessel C was the source of the hemorrhage.

A distribution of the branches of the pudic, similar to that which occurred in this case, is more frequent than is generally supposed. But I had neglected to observe it until a few years ago; my attention having been at that time particularly directed to the varieties in the branches of the pudic, by the proposal made by M. Dupuytren to carry the incision through the prostate in a direction upwards, instead of laterally. There happened to be at that time two bodies in the dissecting-room in Great Windmill-street, in both of which the artery to the penis passed along the prostate. I have always since taken notice of this variety in my public demonstrations. Indeed, after I found that pressure on the main pudic had no effect on the bleeding, I told several of the pupils that I feared there was such a distribution of the vessels in this case.

I have since referred to the splendid work of Tiedmann, which was lately brought to this country. In the thirtieth Plate, there is a figure representing exactly the same distribution of the arteries to the penis as that found on the examination of my patient. As the work is scarce and very expensive, I have given a copy of the drawing. Tiedmann describes it as representing an unusual distribution of the left dorsal artery of the penis, in a man of thirty-six years of age. He adds, in a note, that he has seen the same on both sides in a man of twenty-eight years of age, and also in a boy. He has likewise seen a similar variety in the arteries passing to the clitoris.

***Figura II. Tabula Trigessima.***

**" Conspicitur decursus insolitus arteriæ dorsalis penis sinistræ e cadavere viri triginta sex annorum.**

**" 4. Os Sacrum. 5. Os Pubis. 13. Intestinum Rectum. 14. Vesica Urinaria. 15. Prostata. 18. Crus dextrum Penis abscissum. 19. Hasta Virilis. 21. Ligamentum Suspensorium Penis. 22. Arteria Aorta. 26. Arteria Iliaca communis sinistra. 29. Arteria Hypogastrica. 32. Arteria Glutea. 33. Arteria Ischiadica. 35. Pars Arteriæ umbilicalis in ligamentum mutata. 36. Arteria vesicalis. 37. Truncus pro arteria pudenda communi et hæmorrhoidali media. 40. Arteria Penis quæ insolito modo infra symphysin ossium pubis ad penem decurrit. 44. Arteria Profunda Penis. 45. Arteria Dorsalis Penis. 48. Arteria Dorsalis Penis dextra.***

" Similem casum observavi in utroque latere viri viginti octo annorum, nec non in puero. Tandemque in cadavere virginis octodecem annorum arteriam clitoridis pari modo decurrentem vidi. Burns (p. 350,) narrat se hunc lusum quater vidisse semper in maribus. Veteriores anatomiæ cultores Vesalius, Valverdus, Jac. Sylvius, Bauhinus, Veslingus, Highmorus, Winslowus, aliique hanc dispositionem arteriæ dorsalis penis et clitoridis tanquam normam descripserunt."

I find that Winslow has given the description of the artery to the body of the penis in such a way as to lead us to suppose that he did not consider its passing along the prostate, and under the symphysis of the pubes, as being an unusual distribution. (See Winslow, *Traité des Arteres*, § 249, 250, 251.) Haller also states, that the older anatomists generally describe the pudic as passing along the prostate, and under the symphysis pubis: (see his Fasciculus iv.) Mr. Burns also, in his work on Aneurism, mentions that he has seen a similar distribution in three instances. Dr. Barclay has also given us more authorities for this variety. Within these few days, one of my pupils

* This is from the second figure of the thirtieth Plate of " Friederici Tiedemann, Anatomes et Physiologiæ in Academia Heidelbergensi Professoris, Tabulæ Arteriarum Corporis Humani."

pointed out to me the following passage in the work lately published by Mr. HARRISON, of Dublin, and which I have much satisfaction in quoting.

"In my dissections of the arteries, I have occasionally observed that the pudic artery, on one or both sides, appeared unusually small; and, on more accurate examination in such cases, I have found that the internal iliac had given off a distinct branch, which ran along the side of the bladder and prostate gland, and, passing beneath the arch of the pubes with the dorsal veins, became the dorsal artery of the penis. *Should such a variety exist in one who was to become the subject for the lateral operation of lithotomy, I fear this artery must be wounded;* and, judging from its size and situation in those cases in which I have seen it take this course, I should apprehend very serious consequences from its division. It is not improbable but that some of those alarming and fatal cases of hemorrhage, which have occurred even to the best operators, may have depended on this variety. I may observe, that I have noticed this variety very frequently in children under eight years of age, but as yet I have only met with three instances of it in the adult."*

The above authorities are sufficient to induce us to consider the artery, from which the hemorrhage took place in my patient, as a source of great danger in the operation of lithotomy. I do not purpose to enter at present into an examination of the most likely mode of avoiding this; but I trust that the history of this case will have the good effect of directing the attention of lithotomists to the question. The following passage from the work of the veteran BOYER, although it may be in some degree consolatory to the surgeon who loses a patient by hemorrhage, is rather an appalling testimony of the difficulties and dangers of the operation of lithotomy.

"L'hemorragie est un des accidens les plus ordinaires de la lithotomie. Cet accident a souvent eté mis sur le compte de l'operateur, ou celui de precedé dont il a fait choix. Mais presque toujours injustement, parceque les arteres du perinée offrent dans leur situation et dans leur direction des varietés telles, que le chirurgien le plus habile n'est jamais absolument certain de les eviter, quelque soit le procédé dont il se serve."†

* *Surgical Anatomy of the Arteries.* By R. HARRISON, Demonstrator of Anatomy, Dublin. Vol. ii. p. 124.

† *Traite des Maladies Chirurgicales.* Par M. le Baron BOYER. Tome ix. p. 429. —Paris, 1824.

## Figures and Tables

**Fig 2. Fig 1. f1:**